# Adherence to pediatric diabetic ketoacidosis guidelines by community emergency departments’ providers

**DOI:** 10.1186/s12245-017-0137-8

**Published:** 2017-03-21

**Authors:** Janine E. Zee-Cheng, Emily C. Webber, Samer Abu-Sultaneh

**Affiliations:** 1 Section of Pediatric Critical Care Medicine, Indianapolis, IN USA; 2 Department of Pediatrics, Section of Pediatric Hospital Medicine, Indiana University School of Medicine and Riley Hospital for Children at Indiana University Health, 705 Riley Hospital Drive, ROC 4905, Indianapolis, IN 46202-5225 USA

**Keywords:** Diabetic ketoacidosis, Pediatric, Type 1 diabetes mellitus, Clinical guidelines, Community emergency departments

## Abstract

**Background:**

Diabetic ketoacidosis (DKA) is a common presentation of type I diabetes mellitus to the emergency departments. Most children with DKA are initially managed in community emergency departments where providers may not have easy access to educational resources or pediatric-specific guidelines and protocols that are readily available at pediatric academic medical centers. The aim of this study is to evaluate adherence of community emergency departments in the state of Indiana to the pediatric DKA guidelines.

**Methods:**

We performed a retrospective chart review of patients, age 18 years of age or under, admitted to the pediatric intensive care unit with a diagnosis of DKA.

**Results:**

A total of 100 patients were included in the analysis. Thirty-seven percent of patients with DKA were managed according to all six guideline parameters. Only 39% of patients received the recommended hourly blood glucose checks. Thirty percent of patients received intravenous insulin bolus, which is not recommended.

**Conclusions:**

Non-adherence to pediatric DKA guidelines still exists in the state of Indiana. Further, larger studies are needed to reveal the etiology of non-adherence to pediatric DKA guidelines and strategies to improve that adherence.

## Background

Worldwide, from 1990 to 2008, the incidence of type I diabetes increased from 2.8 to 4.0% per year [1]. Between 2000 and 2008 in the United States, the prevalence of type I diabetes increased by 21.1% [2]. Diabetic ketoacidosis (DKA) is a common presentation of type I diabetes mellitus with one study demonstrating 38.9% of type I diabetes patients presenting in DKA [3]. The biochemical criteria of DKA are hyperglycemia (blood glucose > 11 mmol/L or 200 mg/dL); venous pH < 7.3 or bicarbonate <15 mmol/L; and the presence of ketonemia or ketonuria [4].

Cerebral edema (CE) is a serious complication of DKA with mortality rate between 21 and 24% [5]. This cerebral edema results in chronic central nervous system morbidity in 10-25% of children [6]. Severe CE occurs in 0.5% to 1% of DKA episodes [5, 7, 8], but mild CE, which is associated with minor mental status changes or is asymptomatic, occurs more frequently [9, 10].

A number of risk factors for CE have been identified. Among them are younger age, new onset DKA, administration of large volumes of fluid, insulin administration within the first hour of fluid treatment, administration of sodium bicarbonate, and administration of insulin bolus [11]. Because of these risk factors, the American Diabetes Association (ADA) established guidelines in 2006 for care of pediatric patients with DKA. These guidelines include: initiation of fluid therapy immediately after recognition of DKA, no more than 40 mL/kg of fluid to be given over 2–4 h, only isotonic fluid (such as 0.9% saline or Lactated Ringer’s) to be used for the first 4–6 h, administration of insulin at least 1 h after fluid resuscitation has begun (not concurrently), and use insulin infusion rather than bolus. The guidelines do not recommend administration of sodium bicarbonate [12]. A systematic review of 44 articles examining sodium bicarbonate treatment in DKA found that the evidence does not justify the administration of sodium bicarbonate, especially in the pediatric population, due to possible clinical harm and lack of sustained benefits [13].

Prior to these guidelines, there was significant variability of care among community and referring providers [14], and even 5 years after the guidelines were established, variability of care existed in Illinois [15]. Although these guidelines exist, it is up to individual institutions to establish protocols to standardize patient care.

Pediatric patients with DKA often present first to community emergency departments (EDs) and are then referred to tertiary care centers for ongoing management of their DKA. Prior studies have evaluated variability of care via surveys administered to physicians and providers at referring facilities [15, 16], but there has been little exploration into actual adherence of referring providers to pediatric DKA guidelines.

We hypothesize that despite published guidelines for pediatric DKA management, there remains non-adherence to guidelines by community EDs in the state of Indiana.

## Methods

### Study design and setting

This is a retrospective study that was conducted at Riley Hospital for Children at Indiana University Health pediatric intensive care unit (PICU); a 36-bed unit in a 305-bed, academic, tertiary care children’s hospital. The hospital referral area covers the state of Indiana, as well as some parts of Illinois. This study was approved by the Institutional Review Board at Indiana University School of Medicine.

### Selection of participants

**Fig. 1:**
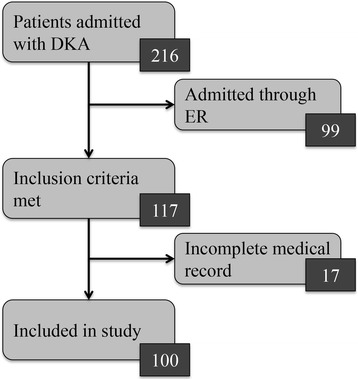
Flowchart demonstrating patient selection. Ninety nine patients were admitted through our emergency department and were therefore excluded from analysis. Seventeen patients were excluded because their medical records were incomplete. One hundred patients were ultimately included in the study

Patients 18 years of age or under who were admitted to the PICU between April 2013 and April 2015 with a diagnosis of DKA were considered for this study. Patients who were admitted through Riley Hospital for Children at Indiana University Health emergency department were excluded. Patients who were first seen at a referring facility and then seen at our emergency department before being admitted to the PICU were also excluded. Patient selection is showed in Fig. 1.

### Methods and measures

Records from referring facilities, transport team records, and PICU admission notes were reviewed. Demographic data including age, gender, new diagnosis of type I diabetes, and presence of insulin pump were collected. The following DKA guideline parameters initiated at the referring facility were collected: administration of intravenous insulin bolus, administration of subcutaneous insulin bolus, administration of fluids in excess of 40 mL/kg over less than 2 h, use of non-isotonic fluids, administration of sodium bicarbonate, and evaluation of blood glucose hourly.

Geographic location of each referring hospital was noted, as was the shortest driving distance between referring hospital and our facility as established by Google Maps. The number of patients from each referring hospital was noted.

### Statistical analysis

Statistical analyses were conducted using STATA 12.1 (College Station, Texas). Mann-Whitney test was performed to evaluate the effect of demographic variables on adherence to all DKA guideline parameters. Mann-Whitney and chi-square tests were performed to evaluate the effect of demographic variables on individual DKA guideline parameters. Logistic regression was performed to establish odds ratio for each demographic variable for adherence to all DKA guideline parameters.

## Results

Between April 2013 and April 2015, 216 patients were admitted to our PICU with DKA. One hundred patients met the study criteria. Demographic information of patients included in the analysis of the study is showed in Table 1.

**Table 1:** Patients’ demographics^a^

Age in years	12 (1.4, 18)
Female	54 (54%)
New diagnosis	54 (54%)
Using insulin pump	10 (10%)

^a^Data are represented as median (25th, 75th IQR) or *n* (%)

**Fig. 2:**
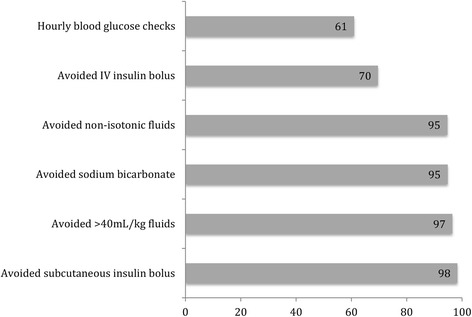
Percentage of patients whose treatment adhered to pediatric DKA management guidelines

A total of 56 hospitals referred patients to Riley Hospital for Children PICU. The average distance that a patient travelled was 62 miles, with a range between 0.4 miles and 164 miles. Of the 100 patients included in the study, only 37 (37%) were managed following all 6 guideline parameters. The majority of referring providers avoided subcutaneous insulin bolus, fluids in excess of 40 mL/kg, non-isotonic fluids, and administration of sodium bicarbonate. However, 30% of patients received an intravenous insulin bolus, and only 61% received the recommended hourly blood glucose checks (Fig. 2).

There was no significant association between non-adherences to the guidelines and patients characteristics (age, gender, new diabetes diagnosis, or using insulin pump). Distance of the referring facility from our facility was not a risk factor for non-adherent to the guidelines.

## Discussion

Despite the establishment of guidelines for treatment of pediatric DKA in 2006, a practice gap still exists. This has been demonstrated previously by Barrios [15] and Clark [16] by a survey of providers caring for patients with DKA, but has not previously been demonstrated by reviewing all patient records of referring facility.

From review of medical records of patients transferred from referring hospitals to our PICU, it is evident that non-adherence to guidelines remains an issue. Only 37% of patients transferred to the PICU from referring hospitals met all six DKA guideline parameters, and less than two thirds of patients received recommended hourly blood glucose evaluation. Therapies that are not recommended, such as administration of IV insulin bolus and sodium bicarbonate, are still being done.

Children with DKA seen in non-pediatric community hospital’s EDs are often managed by adult trained emergency physicians. Guidelines for the management of adult diabetic ketoacidosis still include administration of IV insulin bolus and sodium bicarbonate [17]. Because referring facilities treat a higher percentage of adults than children, it may be speculated that this is the reason that IV insulin and sodium bicarbonate was administered to pediatric patients referred to the PICU. From the statistical analyses performed, there did not appear to be any factors that increased the likelihood that all guideline parameters would be met.

In the planning of this study, it was postulated that geographic distance from an academic center might be a risk factor for receiving care that was non-adherent to guidelines. It was thought that more rural centers, who do not have access to the number of learners or the frequent didactic session that are the hallmark of university-affiliated facilities, might be more likely to be non-adherent to guidelines. However, this study did not demonstrate any relationship between distance and adherence to guidelines. A practice gap exists, no matter how rural or urban the setting. The effect of distance on pediatric DKA management maybe better characterized with future larger studies.

The two guideline parameters in which non-adherence occurs most frequently are administration of intravenous insulin bolus and failing to check hourly blood glucoses. Further, larger studies are needed to reveal the etiology of non-adherence to pediatric DKA guidelines and strategies to improve that adherence.

### Limitations

This study was limited by its retrospective nature. It was also limited by the relatively small number of patients. Study of a larger number of patients may have revealed more of an effect of patients’ demographic variables on adherence to guidelines. We did not collect data about the emergency care physicians training and maintenance of certification. A larger study would be able to look at the relationship between physician training and adherence to the pediatric DKA guidelines. Additionally, because of the number of patients and the relatively low incidence of cerebral edema, clinically significant cerebral edema was not demonstrated in any patient. Outcome data, such as length of PICU stay, time to correction of DKA, and length of need for insulin drip, were not collected. Other limitations include: the possibility of incomplete transport or referring facility records not evident on chart review, i.e., if blood glucoses were checked on a glucometer but not documented by the referring facility.

### Educational tool

In response to the practice gap found in this study, a free, online educational tool was created online at http://www.pedsDKA.com. This tool was created because the authors believe that access to medical education and to current guidelines should be available to all practitioners, regardless of geographic location or academic presence. Since non-adherence to guidelines was found statewide, it was clear that additional education was required in all referring facilities. The tool consists of a case scenario with three questions that are representative of the guidelines, as well as a reference section that contains recommendations for each of the guideline parameters. It is intended for referring providers, both in the emergency department and primary care settings. A link to the tool was emailed to emergency department directors of referring hospitals in the state of Indiana. It was also distributed via social media networks, such as Twitter.

## Conclusions

Non-adherence to pediatric DKA guidelines exists among community emergency departments’ providers in the state of Indiana. The two guideline parameters in which non-adherence occurs most frequently are administration of intravenous insulin bolus and failing to check hourly blood glucoses. Further, larger studies are needed to reveal the etiology of non-adherence to pediatric DKA guidelines and strategies to improve that adherence.

## Abbreviations

DKA: Diabetic ketoacidosis
EDs: Emergency departments
PICU: Pediatric intensive care unit
